# Long-term safety and efficacy outcomes of the Acellular Tissue Engineered Vessel (ATEV) in extremity arterial trauma repair

**DOI:** 10.1016/j.jvscit.2025.102042

**Published:** 2025-11-04

**Authors:** Michael A. Curi, Ernest E. Moore, Nicholas Namias, Rishi Kundi, Ying Wei Lum, Charles J. Fox, Ilya Goldin, Sophia Bou-Ghannam, Mauricio Berdugo, Zakaria Khondker, Shamik Parikh, Laura E. Niklason

**Affiliations:** a Division of Vascular Surgery, Rutgers-New Jersey Medical School, Newark, NJ; b Department of Surgery, Ernest E. Moore Shock Trauma Center at Denver Health, University of Colorado, Denver, CO; c Daughtry Family Department of Surgery, Miller School of Medicine, University of Miami, and Ryder Trauma Center, Jackson Memorial Hospital, Miami, FL; d Division of Vascular and Endovascular Trauma Surgery, R. Adams Cowley Shock Trauma Center, University of Maryland School of Medicine, Baltimore, MD; e Johns Hopkins Bayview Medical Center, Johns Hopkins Hospital, Baltimore, MD; f Department of Surgery and Hemodialysis Access Center, Shaare Zedek Medical Center, Jerusalem, Israel; g Humacyte Global Inc, Durham, NC

**Keywords:** Bioengineered artery, Prospective study, Regenerative, Vascular conduit, Vascular graft

## Abstract

**Background:**

In cases of arterial injury, use of autologous vein for arterial repair is sometimes not feasible. We previously reported the short-term (ie, 30-day) outcomes of the Acellular Tissue Engineered Vessel (Symvess) in treating extremity vascular injuries. This study evaluates the long-term safety, efficacy, and mechanical durability of Symvess in patients with arterial trauma.

**Methods:**

This prospective analysis focused on patients from the CLN-PRO-V005 clinical study who sustained noniatrogenic vascular injuries to the extremities, and for whom saphenous vein was not feasible for use. Patients received Symvess and were followed for up to 36 months. Primary outcomes include graft patency, limb salvage, adverse events, and mechanical durability, as assessed through clinical evaluation, imaging, and independent adjudication. Long-term patency, limb salvage, patient survival and infection rates were analyzed by the Kaplan-Meier method.

**Results:**

This study evaluated 54 patients who underwent extremity vascular repair with Symvess and were followed for up to 36 months. Mechanisms of injury were primarily penetrating (57%) or blast-related. Follow-up ranged from 1 day to 42 months (median, 10.7 months), totaling 59.5 patient-years. Primary patency was 58.3%, and secondary patency was 65.7% at month 12; limb salvage at month 12 was 87.3%. Infection-free rates remained at 92.9% from months 3 through 36, with no infections after day 37. All deaths (n = 6; 11.1%) occurred within the first 6 months. No deaths or amputations were attributed to the Symvess conduit. Adverse events and serious adverse events declined over time, and no cases of immune-mediated rejection were observed. There were no cases of unprovoked, spontaneous Symvess rupture or mechanical failure.

**Conclusions:**

Symvess use for arterial reconstruction in setting of limb threatening arterial injury is associated with low infection rates, and acceptable limb salvage outcomes over 36 months in a high-risk trauma population when use of saphenous vein was not feasible. These results suggest Symvess represents a new alternative when autologous vein is not feasible for arterial reconstruction in extremity trauma.

Current clinical guidelines for the management of traumatic vascular injuries emphasize the use of autologous vein grafts as the first-line conduit due to their long-term durability and resistance to infection, a principle first demonstrated by Kunlin in 1951.^1^ Civilian registry data, such as the Prospective Observational Vascular Injury Treatment (PROOVIT) registry, report excellent outcomes with vein grafts, including in-hospital patency exceeding 90%, and low infection rates.^2^ However, autologous vein is not always feasible; its use requires time-intensive harvesting that can delay revascularization in urgent settings, and its availability may be limited in patients with multiple injuries or with poor vein quality.^3^

The guidance states that when autologous vein is not feasible, then synthetic grafts may be utilized.^4^ First introduced in the 1950s, synthetic conduits are readily available and avoid the need for vein harvest.^5^ However, their use in the trauma setting is constrained by high infection risk, particularly in contaminated wounds containing foreign material, devitalized tissue, or open fractures.^6^, ^7^, ^8^ In more recent decades, alternative biological conduits, such as cryopreserved allografts and xenografts, have been explored but suffer from complications like dilatation, calcification, and spontaneous rupture, limiting their widespread clinical use in trauma.^9^^,^^10^

Although the published vascular trauma literature comprises robust retrospective registries, particularly from wartime experiences,^11^^,^^12^ as well as single-center experiences, the follow-up is often confined to the perioperative period, with little to no prospective long-term data. The PROOVIT registry, although the largest prospective registry for vascular trauma in the world, is mostly confined to the acute inpatient period.^2^

The recent United States Food and Drug Administration (FDA) approval of the Acellular Tissue Engineered Vessel (ATEV, Acellular Tissue Engineered Vessel-tyod, or Symvess) for the treatment of extremity vascular trauma represents the first novel biologic vascular conduit approval in years, and specifically for the vascular trauma indication.^13^ Symvess is a bioengineered human vessel that is available “off-the-shelf” and is designed to repopulate with the patient’s own cells over time. In trauma patients, Symvess may offer an alternative to synthetic vascular grafts, which are associated with high infection and failure rates in contaminated or injured fields.^14^^,^^15^

The prospective single-arm phase II/III study, CLN-PRO-V005 (CLiNical PROgram Vessel), enrolled adult vascular trauma patients requiring arterial reconstruction in the United States and Israel for whom repair with autologous vein was deemed not feasible and who would have otherwise received a synthetic or alternative conduit. Consented patients received the ATEV (Symvess) for arterial repair.^13^ All efficacy and safety endpoints were prospectively collected, with primary analysis at 30 days and extended follow-up for up to 3 years. Initial results from the V005 study were published in 2024,^16^ focusing on safety and efficacy outcomes at 30 days in patients with extremity vascular trauma.

The demographics of the original V005 cohort (n = 51) were reported previously.^16^ This publication includes an additional three patients enrolled after the original data cutoff of June 30, 2023 (n = 54 total), and builds upon the initially reported short-term results by presenting extended follow-up of patients treated with Symvess for extremity vascular trauma. These patients comprised the FDA-specified extremity cohort undergoing urgent repair of noniatrogenic vascular injuries, and formed the basis for FDA approval of Symvess.^13^ The data cutoff for this report is April 10, 2025. This present analysis provides additional insight into the long-term performance of Symvess in traumatic vascular repair, including patency, limb salvage, infection, patient survival outcomes, and conduit durability. This prospective, long-term analysis of a novel vascular conduit is the first of its kind in the field of vascular trauma.

## Methods

### CLN-PRO-V005 trial

The CLN-PRO-V005 trial is a prospective, multicenter, open-label, single-arm phase II/III study designed to evaluate the safety and efficacy of the ATEV in patients with life- or limb-threatening vascular trauma, in whom repair with autologous vein was not feasible. Conducted at 17 Level 1 trauma centers across the United States and two centers in Israel from September 2018 through August 2023, the trial enrolled patients who required vascular replacement or reconstruction due to arterial trauma. The primary efficacy endpoint was primary patency at 30 days postimplantation. Secondary endpoints included secondary patency, limb salvage, conduit infection rates, and overall patient survival, all assessed at the 30-day mark. Follow-up evaluations were scheduled at day 5, day 30, and months 3, 6, 9, and 12 after implantation, with continued assessments every 6 months up to 36 months. This trial is registered with ClinicalTrials.gov, NCT03005418.

#### V005 safety reporting

Safety analyses were conducted on the cohort of patients with noniatrogenic extremity injuries who were treated with Symvess, which is the focus of this manuscript. Incidence of reported aneurysm formation (both true aneurysms and pseudoaneurysms), anastomotic bleeding or spontaneous rupture of the conduit, Symvess infection, and removal were recorded separately from other adverse events (AEs) and listed by visit and for the overall study. Events of thrombosis or stenosis were determined by the investigator and reported as AEs. AEs considered by the investigator to be “definitely” or “possibly” related to the conduit were recorded in terms of incidence, severity, and time to onset and duration.

Symvess infection was determined by the investigator and was based on patient symptoms, clinical assessment, and laboratory findings including culture of microbes from wound or blood samples. Explanted grafts were further examined for any bacterial infiltration into the vessel wall, along with histopathological staining. It was mandatory in this study for all patients to have received at least at least 1 day of antibiotic prophylaxis initiated on the day of the surgery. Longer antibiotic prophylaxis was left at the discretion of the investigator.

A Data Monitoring Committee was assigned to review safety on an ongoing basis and provided recommendations about stopping, continuing, or otherwise modifying the study. The Data Monitoring Committee consisted of individuals who were not directly involved in the conduct of the study. Additionally, and as previously described,^4^ an independent Adjudication Committee (AC) composed of vascular surgeons experienced in the surgical management of vascular trauma was established to assess the intercurrent events occurring before the day 30 milestone.

#### Trauma severity scoring

As part of clinical data collection, all patients were assessed for injury severity by the treating surgeon using standardized trauma scoring systems. The Injury Severity Score (ISS) was calculated to quantify overall injury burden across multiple anatomical regions. The Abbreviated Injury Scale (AIS) was used to classify the severity of specific injuries, including vascular and soft tissue damage. In addition, the Mangled Extremity Severity Score (MESS) was recorded to evaluate the extent of extremity trauma and guide limb salvage decision-making. These scores were used to characterize the clinical presentation of patients at the time of enrollment.

### Ultrasound assessment

Duplex ultrasound of the entire Symvess conduit was obtained at scheduled follow-up visits through month 36 to assess the inner diameter of the conduit over time. Inner diameter measurements were obtained at the midpoint of the implanted conduit. Investigators used standard protocols for vascular access surveillance. Vessel patency and diameter stability were assessed to detect any trends toward dilatation or narrowing over time. Diameter measurements were recorded at day 30, and at 3-, 6-, 9-, 12-, 24-, and 36-month follow-up visits, where available.

### Statistical analysis

The primary and key secondary endpoint assessment was at 30 days. The statistical methodology, including the prespecified while-on-treatment strategy, the adjudication of intercurrent events by an independent adjudication committee, and the handling of missing data at day 30, have all been previously presented.^16^

Long-term outcomes were evaluated by the Kaplan-Meier method at 1-, 2- and 3-year time points, with patient censoring for intercurrent events unrelated to the outcome of interest. Censoring was applied for death not related to the conduit or for loss to follow-up.^17^^,^^18^ Time-to-event analyses were conducted for 36 months of follow-up using all available data as of the April 10, 2025, data cutoff for secondary patency, limb salvage, conduit infection, and patient survival.

Safety data were collected for up to 36 months for patients continuing to be available for follow-up. Data on Symvess infection, removal, rupture, and bleeding were collected in addition to AEs, serious AEs, and mortality in the safety follow-up. Safety events were reported in terms of number of events and number and percentage of patients experiencing an event and grouped into early events (up to 1 year) vs late events (beyond 1 year) to clearly distinguish safety events related to initial injury and shorter-term complications vs longer-term conduit performance.

## Results

### Overview of patients analyzed

The CLN-PRO-V005 trial enrolled a total of 72 patients through the end of study enrollment, of whom 54 comprised the noniatrogenic extremity injury population. Among these 54 patients, 42 had lower extremity injuries and 12 had upper extremity injuries, with some patients sustaining injuries to more than one limb/vessel. As per the prespecified enrollment criteria, use of autologous vein was not feasible in these patients in the judgement of the treating surgeon. The most common reasons for autologous vein repair not being feasible were poor vein quality, prior vein harvest, concomitant venous injuries, or urgency of repair that precluded the harvesting of the autologous vein. Additional reasons included technical challenges such as obesity, external fixation of the limb, and contamination. The mean ISS for the 54 patients was 21.1. The mean MESS was 5.1 ± 1.8, and one-quarter of the patients had MESS ≥6. By the AIS, severe or critical injuries were reported for 74% of the patients. Patient demographics and injury characteristics for this cohort are presented in Table I.

**Table I:** CLN-PRO-V005 extremity patient demographics and injury characteristics

Characteristic	V005 (n = 54)
Race	
Black or African American	26 (48.1)
White	23 (42.6)
Other	5 (9.3)
Sex	
Male	40 (74.1)
Female	14 (25.9)
Age, years	33.4 (13.6)
Mechanism of injury	
Penetrating	31 (57.4)
Blunt	23 (42.6)
ISS	21.1 (10.2)
MESS	5.1 (1.8)
AIS	
Serious	14 (25.9)
Severe	26 (48.1)
Critical	14 (25.9)

*AIS,* Abbreviated Injury Scale; *ISS,* Injury Severity Score; *MESS,* Mangled Extremity Severity Score.

Data are presented as number (%) or mean (standard deviation).

### Long-term outcomes

As of April 10, 2025, 23 (43%) and nine (17%) patients had completed 12 and 36-month follow-up, respectively. An additional seven patients (13%) were ongoing beyond 12 months. Duration of follow-up ranged from 1 day to 42 months, with a mean of 13.3 months (Kaplan-Meier estimated median 10.7 months), for a total of 59.5 patient-years of follow up. Primary reasons for study discontinuation included loss to follow-up (n = 15; 28%), Symvess removal (n = 8; 15%), death unrelated to Symvess (n = 6; 11%), or withdrawal of consent (n = 3; 6%).

**Fig 1:**
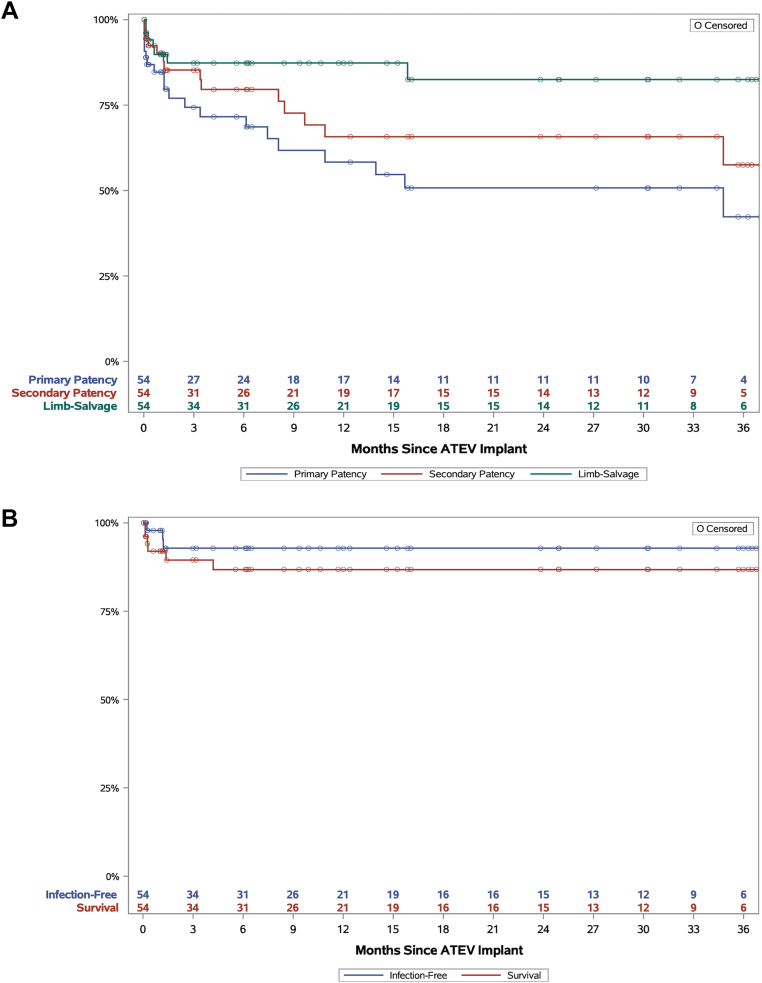
Kaplan-Meier plots of extremity trauma patients who received Symvess, for primary patency, secondary patency, and limb salvage **(A)**; and freedom from infection and patient survival **(B)**. For primary and secondary patency, the standard error of the Kaplan-Meier estimate exceeds 10% after month 34; for limb salvage, infection and survial, the standard error did not exceed 10%. *ATEV*, Acellular Tissue Engineered Vessel.

Kaplan-Meier estimated long-term outcomes are provided in Fig 1 and are summarized in Supplementary Table I (online only). Primary patency declined gradually over the first 15 months and stabilized thereafter, whereas secondary patency declined gradually over the first 10 months and stabilized thereafter (Fig 1, *A*). Secondary patency was 65.7% at month 12 and 57.5% at month 36. Of the 54 patients treated, six lost secondary patency within the first 30 days (Fig 1, *A*).

Eight patients who maintained 30-day patency subsequently lost secondary patency. Of these eight patients, three required revascularization, and five retained the occluded Symvess with no further intervention due to well-established collateral circulation; all eight patients retained their limbs for the duration of follow-up. In the three revascularization patients, autologous vein had not been used for the initial repair due to patient preference in one case, poor vein quality in another, and challenging harvest conditions related to fixation procedures at the time of surgery in a third. The revascularization procedures included an axillary-to-brachial bypass due to gradual Symvess occlusion and inconsistent antiplatelet (ASA) use, a superficial femoral-to-posterior tibial bypass for lower extremity Symvess thrombosis, and a popliteal-to-proximal tibial bypass following graft erosion and bleeding due to deep wound infection (Supplementary Appendix, case 3 [online only]). In three cases of loss of secondary patency after month 12, patients reportedly had stopped or were inconsistently using prescribed ASA therapy.

Limb salvage rate was 87.3% at month 12 and 82.5% at month 24 (Fig 1, *A*). There were seven cases of limb loss, of which five occurred prior to day 30. None of these five cases were attributed to the Symvess conduit but rather to concomitant injuries and the mangled extremity. One amputation of the treated limb occurred on day 43 following overlying muscle necrosis that exposed the conduit with consequent Symvess microbial contamination. Investigator-determined reperfusion injury and extensive infection then necessitated amputation.

One amputation occurred after month 12. The patient had a superficial femoral artery to below-knee popliteal Symvess graft for a gunshot wound. The patient self-discontinued aspirin and presented on day 423 with acute limb ischemia and graft thrombosis. He underwent catheter-directed thrombolysis and then developed emboli to the foot. This patient underwent a Chopart amputation on day 457, followed by a below-knee amputation on day 482. The Symvess conduit was proximal to the amputations, was confirmed to be patent, and required no further interventions. The patient completed the study with a patent Symvess in place.

There were a total of three conduit infections, with one occurring prior to day 30 and the other two occurring shortly thereafter. The infection-free rate remained stable at 92.9% from months 3 through 36 (Fig 1, *B*). Across the entire follow-up period, 27 wound/surgical site infections were reported, but only these three extended into the Symvess graft itself.

Of the six patient deaths (11.1%) that occurred in this cohort, four occurred within 30 days. The patient survival rate stabilized after month 4 at 86.6% through month 36 (Fig 1, *B*). None of the deaths were attributed to Symvess, as determined by the independent AC.

Briefly, one patient died from complications of severe thoracoabdominal trauma sustained in a motorcycle accident, requiring extracorporeal membrane oxygenation support before succumbing on day 4 after injury. Another patient died from acute respiratory distress syndrome and multiple organ failure, unrelated to the vascular repair or study procedure, also on day 4. A third patient died of cardiopulmonary arrest related to methicillin-resistant *Staphylococcus aureus* sepsis and necrotizing pneumonia following multiple gunshot wounds on day 8. A fourth patient who was on intensive supportive care succumbed on day 9 to complications of septic shock and multiorgan failure, following extensive orthopedic trauma and bowel ischemia with necrosis. After day 30, one patient died on day 42 from complications of multiple gunshot wounds to the head upon exiting the hospital on day 39. Another patient died on day 128 of acute overdose that was deemed unrelated to Symvess or to the index surgical procedure.

### Long-term mechanical durability

**Fig 2:**
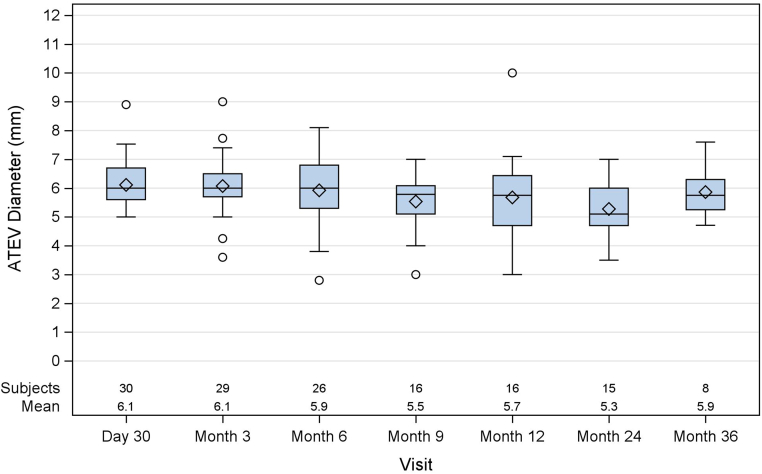
Duplex ultrasound assessment of mid-graft diameter. Mean diameters (*diamonds*) are reported for each scheduled follow-up evaluation. The *boxes* represent the interquartile range, and the *diamonds* and *horizonal lines* inside each box denote the mean and median mid-graft Acellular Tissue Engineered Vessel (*ATEV*) diameter, respectively, at each time point. The *whiskers* extend to 1.5 times the interquartile range, which is the range between the first quartile (25th percentile) and the third quartile (75th percentile) of the data. Each *circle* represents a data point outside the whisker.

To assess mechanical durability and stability of the conduit, duplex ultrasound was used to evaluate patency and vessel inner-to-inner wall diameter at each follow-up visit through month 36. Fig 2 summarizes ultrasound data for vessel diameter at the midpoint of the conduit. Among 23 patients followed beyond 12 months, average mid-graft vessel diameter did not notably depart from the baseline of 6 mm, measuring 5.7 mm, 5.3 mm, and 5.9 mm at 12, 24, and 36 months, respectively. No trend of either dilatation nor narrowing was observed for Symvess diameter out to 36 months.

### Long-term safety outcomes related to Symvess

Important safety events were evaluated through 36 months and included thrombosis, conduit infection, true and pseudoaneurysm, conduit rupture (ie, bleeding or anastamotic failure), and death (Table II).

**Table II:** Important safety events over 36 months

	Year 1		Years 2-3		All	
	No. (%)	Events	No. (%)	Events	No. (%)	Events
Thrombosis	14 (25.9)	20	3 (5.6)	4	17 (31.5)	24
Stenosis	6 (11.1)	10	2 (3.7)	3	8 (14.8)	13
Surgical site/wound infections	17 (31.5)	26	1 (1.9)	1	18 (33.3)	27
Symvess infection	3 (5.6)	3	0	0	3 (5.6)	3
Pseudoaneurysm	2 (3.7)	2	0	0	2 (3.7)	2
Aneurysm	1 (1.9)	1	0	0	1 (1.9)	1
Symvess rupture or anastomotic failure^a^	4 (7.4)	4	0	0	4 (7.4)	4
Death	6 (11.1)	6	0	0	6 (11.1)	6

a Bleeding from the anastomotic site.

A total of 24 graft thromboses were reported in 17 patients over the duration of the follow-up. Fourteen patients experienced 20 thrombotic events within the first year, including nine events that occurred within 30 days (eight of which were reported within 1 week). Among these patients, nine had adequate collateral circulation and required no intervention, with the thrombosed Symvess left in place. Two patients were successfully treated by thrombectomy with Symvess retained, and two others underwent bypass surgery with Symvess left in place. In the remaining four patients, Symvess was removed and replaced with another graft type in three cases, while removal with subsequent amputation was performed in one previously described case at day 43; full details of this case are provided in the Supplementary Appendix, patient 1 (online only) (Table II). Thirteen stenosis events were reported in eight patients over the study period. Four patients with thrombosis also had ultrasound-confirmed anastomotic stenosis (Table II).

Within the first year, 26 surgical site or wound infections were reported in 17 patients, with one additional event being reported beyond 12 months (Table II). In the setting of 27 total wound infections reported, there were three Symvess conduit infections reported over the 36-month follow-up. The first occurred on day 7 in the context of crush injuries and deep tissue involvement requiring fasciotomies following a motor vehicle accident. In this case, Symvess infection occurred in the context of a necrosed and infected muscle graft. The second Symvess infection was reported on day 36 in a patient who sustained bilateral injuries with open fractures and extensive soft tissue damage following a motor vehicle accident; the overlying skin graft had necrosed and associated wound infection extended into the conduit. A third infection was reported on day 37 in a patient with significant wound contamination and soft tissue necrosis following an industrial accident involving a degloving injury and multiple fractures. Necrosis of the overlying muscle graft resulted in graft exposure with subsequent bleeding. The clinical contexts of these three cases are further detailed in the Supplementary Appendix (online only; see case 1 [day 37], case 2 [day 7], and case 3 [day 36]). The three conduit infections involved a range of organisms: *Staphylococcus* species including *S. epidermidis* and diphtheroids, gram-negative rods with inflammatory cells, and *Stenotrophomonas maltophilia* with *S. epidermidis.*

In the 54-patient cohort, one case of pseudoaneurysm was reported on day 19, developing at the distal anastomosis in the setting of severe wound infection and surrounding tissue necrosis/breakdown. After a sentinel bleed, the Symvess was clamped and subsequently ligated after hemorrhage. The second case of pseudoaneurysm occurred on day 73, developing near the distal anastomosis adjacent to orthopedic screws placed to fixate a fracture. On the same day, the pseudoaneurysm was resolved by stenting without impacting two-vessel distal runoff. The patient remained on study for 725 days. A single report of mid-graft aneurysmal dilation was reported at month 6. This was the previously described patient who developed graft thrombosis at day 423.

Secondary hemorrhage due to compromised graft integrity was reported in four patients (Supplementary Table II and Supplementary Appendix, online only). Bleeding was the presenting symptom in these cases. These events were heterogeneous in timing and presentation, but in no case was spontaneous, unprovoked Symvess rupture observed. No cases of limb loss or patient death were deemed attributable to Symvess per se by the independent AC. Specifics of each case of Symvess bleeding or anastamotic failure are detailed in the Supplementary Appendix (online only).

### Other safety observations

Across all implanted patients in the V005 study, there were no clinical indications of immune-mediated rejection of Symvess in any patient during follow-up. We observed no difference in clinical outcomes of limb salvage or infection by graft length, although lower patency in longer (more than median implanted length of 7.5 cm) grafts may reflect confounding effects of injury severity.

## Discussion

Symvess is a first-in-class bioengineered vascular conduit of human origin available for off the shelf use, and has recently received FDA approval for use in urgent arterial extremity trauma repair when repair with autologous vein is not feasible. The original report of the V005 study focused on 30-day outcomes in 51 patients who had no feasible autologous vein options, as of a data cutoff of June 30, 2023.^16^ This report presents long-term outcomes for follow-up of patients with noniatrogenic extremity injury who were enrolled in the CLN-PRO-V005 study, including three additional patients not included in the original report. The study cohort demonstrated demographics and injury characteristics consistent with general patterns observed in other vascular trauma populations (Table I).

This complex cohort of patients with extremity trauma did not have a feasible autologous conduit option for repair at the time of injury, and many presented with high MESS indicating elevated risk of limb loss, high ISS indicating severe trauma, and contaminated wounds. These patients in general had more severe and complex injuries, along with a higher percentage of blunt or crush injuries, than those typically treated with the first-line treatment option of autologous vein.^19^ As is often noted in arterial trauma settings,^20^, ^21^, ^22^ the majority of adverse patient outcomes were reported in the first 30 days and were associated with the severity and/or complications of the initial injury. Rates of conduit infection, limb salvage, and patient survival plateaued after 3 months, once early complications were resolved, and remained relatively constant through the 3 years of follow-up (Fig 1).

A single-size Symvess conduit, engineered with a 6-mm internal diameter and 40 cm of usable length, was available for vascular repair in this study, with one conduit used per initial case.

Symvess demonstrated consistent dimensions and structural integrity over time. Ultrasound imaging showed no trend toward conduit dilation or stenosis through 36 months, with average mid-graft diameters remaining close to the engineered 6 mm across all follow-up intervals (Fig 2).

In the extremity trauma cohort, rupture was an infrequent but serious complication, occurring in four of 54 patients (Table II). Detailed case reports describing the clinical context and contributing factors for each Symvess rupture are provided in the Supplementary Appendix (online only). Importantly, these bleeding events were confined to the postoperative period and were closely associated with hostile local wound conditions, and with infection of the Symvess conduit in three of four cases. Each case of rupture was characterized by at least one, and often several, contributing factors, including necrosis of the overlying skin and soft tissue, incomplete tissue coverage leading to exposure of the conduit, and the presence of localized infection or abscess formation. These complex and multifactorial wound environments underscore the challenges of managing vascular repair in contaminated and complex extremity injuries. Ensuring proper tissue coverage postimplantation is essential to minimize exposure-related complications. Importantly, no unprovoked or spontaneous Symvess structural or mechanical failure was observed. All ruptures occurred in the setting of a complex, failing traumatic wound. As with implantation of any vascular graft, healthy tissue coverage is paramount, and patients should be monitored for bleeding due to graft rupture or anastomotic failure.^13^

The safety evidence for various conduits in vascular trauma remains limited. Military and civilian retrospective studies report autologous vein rupture or “blowout” rates of 2% to 6%, with times occurring around 14 days.^23^ However, these studies are often constrained by fragmented or incomplete AE reporting and small sample sizes, which may underestimate true failure rates. For synthetic grafts, published data are even more limited. One study reports a rupture rate of 12.5% (1 of 8 patients) for PTFE in trauma.^24^ Additional insight comes from the Manufacturer and User Facility Device Experience database (MAUDE, accessdata.fda.gov), maintained by the FDA’s Center for Devices and Radiological Health. Although voluntary and noncomprehensive, the MAUDE database includes over 1000 reports of synthetic graft failures from 2015 to 2025, with more than 10% involving synthetic grafts used to treat vascular trauma. Among these MAUDE reports for synthetic grafts, over 15% cite bleeding or rupture, and roughly 15% are associated with patient death.

To date, the ATEV has been implanted in over 600 patients across nine clinical trials, totaling more than 1200 patient-years of exposure across all indications. No spontaneous ruptures have been reported in trauma patients or in studies for dialysis access or peripheral arterial disease. In addition to the treatment of civilian trauma, long-term follow-up of Ukrainian wartime patients who were treated with Symvess, and in whom 30-day data were also reported in 2025, is currently in press.^25^

Although a randomized controlled trial comparing Symvess with other vascular conduit options such as synthetic grafts and/or cryovein would have been ideal to evaluate performance, conducting such a trial in this population poses logistical and ethical challenges. The acute and unpredictable nature of traumatic vascular injuries often necessitates rapid decision-making in life- and limb-threatening scenarios, leaving little opportunity for randomization. Additionally, clinical judgment frequently dictates conduit selection based on factors such as wound contamination, time constraints, and patient-specific anatomical or physiological limitations, making standardized random allocation difficult.

Although cryopreserved autogenous saphenous vein and femoral artery grafts remain commonly used biologic alternatives in vascular trauma, these conduits lack high-quality prospective data with long-term follow-up. In contrast, the present trial represents the first-ever prospective evaluation of a vascular conduit in trauma, demonstrating Symvess durability and stability over 36 months of follow-up.

This study has limitations. Although it provides long-term safety data for Symvess in a prospective cohort of 54 patients, 15 patients (27.8%) were lost to follow-up, which is a frequent challenge in vascular trauma studies.^26^ Of the 15 patients lost to follow-up, six were lost within the first 3 months, five between months 3 and 12, and four were lost after month 12. Although hard outcomes such as amputations, and relevant safety events such as thromboses or infections were documented, detailed assessments on any long-term functional impairment were not performed. As a relatively new, FDA-approved vascular conduit option now available to surgeons, the real-world follow-up of trauma patients who are treated with Symvess will promote additional understanding of its long-term durability and overall performance in the trauma setting.

## Conclusion

The Symvess conduit appears to be a durable and reasonable long-term alternative for arterial repair in extremity trauma when repair with autologous vein is not feasible. In this prospective cohort, the conduit demonstrated a low infection rate, expected limb salvage outcomes, and no cases of spontaneous rupture over extended follow-up. These findings support the use of Symvess as a viable option in high-risk vascular trauma settings when the use of autologous vein is not feasible.

## Funding

This clinical study was supported by Humacyte Global Inc. The sponsor participated in the design and conduct of the study, the collection, management, analysis, and interpretation of the data, as well as the preparation, review, and submission of the manuscript.

## Disclosures

M.A.C., E.E.M., N.N., R.K., Y.W.L., C.J.F., and I.G. received research support from Humacyte, the sponsor of the study reported in this publication. S.B.G., M.B., and Z.K. are employees of Humacyte and receive a salary and own stock. S.P. is CMO of Humacyte and receives a salary and owns stock. L.E.N. is founder and CEO of Humacyte, member of the Board, receives a salary, owns stock, and has multiple patents either owned or licensed by Humacyte.
