# Supplementary material for: Long-term safety and efficacy outcomes of the Acellular Tissue Engineered Vessel (ATEV) in extremity arterial trauma repair
Source: J Vasc Surg Cases Innov Tech. 2025 Nov 4;12(1):102042. doi: 10.1016/j.jvscit.2025.102042 (PMC12720075; doi:10.1016/j.jvscit.2025.102042)
Supplement: Supplemental Tables 1 and 2 (online only) [file mmc1.docx]

**Supplemental Table 1. CLN-PRO-V005 Kaplan-Meir estimated outcomes for extremity cohort.**

|  | Primary Patency | Secondary Patency | Infection-Free Rate | Limb Salvage Rate | Patient Survival Rate |
| --- | --- | --- | --- | --- | --- |
| Day 30 | 84.6% | 90.1% | 97.9% | 89.8% | 92.0% |
| Month 3 | 74.4% | 85.3% | 92.9% | 87.3% | 89.5% |
| Month 6 | 71.6% | 79.6% | 92.9% | 87.3% | 86.8% |
| Month 12 | 58.3% | 65.7% | 92.9% | 87.3% | 86.8% |
| Month 24 | 50.8% | 65.7% | 92.9% | 82.5% | 86.8% |
| Month 36 | 42.3% | 57.5% | 92.9% | 82.5% | 86.8% |

**Supplemental Table 2. Clinical characteristics of four extremity patients with Symvess failures**.

| **Case** | **Age (yr)** | **Sex** | **Country** | **Injury Type** | **Diagnosis of Vascular Injury** | **Failure day** | **Adjudicated Causes of**  **Symvess Failure** | **Final**  **Status** |
| --- | --- | --- | --- | --- | --- | --- | --- | --- |
| **1** | 18 | M | USA | Blunt | Transection right axillary-brachial artery junction | 43 | Necrosis overlying muscle, subsequent exposure of conduit, rupture | Study discontinuation |
| **2** | 65 | M | USA | Blunt | Pedestrian with MV collision –popliteal artery | 8 | External fixation, inadequate tissue coverage, multiple interventions, wound infection | Symvess removal |
| **3** | 20 | M | Israel | Blunt | MVA popliteal artery injury | 36 | Wound infection, necrotic skin graft coverage of conduit | Symvess removal |
| **4** | 24 | M | USA | Penetrating | Gunshot wound: upper chest, left axillary artery | 19 | Wound infection of surrounding soft tissue, pseudoaneurysm rupture at anastomosis | Lost to follow-up after month 6 visit |
