# Supplementary material for: Long-term safety and efficacy outcomes of the Acellular Tissue Engineered Vessel (ATEV) in extremity arterial trauma repair
Source: J Vasc Surg Cases Innov Tech. 2025 Nov 4;12(1):102042. doi: 10.1016/j.jvscit.2025.102042 (PMC12720075; doi:10.1016/j.jvscit.2025.102042)
Supplement: Supplementary Appendix (online only) [file mmc2.docx]

**APPENDIX**

*Symvess Rupture Case Reports*

The clinical characteristics of four extremity patients who experienced Symvess complications, including disruption, rupture, bleeding or dehiscence, as part of the CLN-PRO-V005 are summarized in **Supplemental Table 2**. Informed consent for the publication of research details and clinical images was obtained from all participants. The following case reports provide a detailed account of each case based on clinical reporting from the operating surgeon/principal investigator, and/or evaluations from an independent, expert adjudication committee.

**Case 1**

An 18-year-old male sustained a severe right arm injury while operating an auger at work. The mechanism of injury involved violent rotation of the arm, resulting in near-complete amputation and 180-degree rotation of the upper arm. Injuries included avulsion of the ulnar artery at the axillary-brachial junction, multiple open fractures, severe muscle destruction, degloving, and nerve injury. The patient was classified at high risk for wound infection and limb loss. Though the injury could have met exclusion criteria, limb salvage was attempted due to the patient's youth and occupation as a manual laborer.

The surgeon determined that no suitable autologous vein was available, and Symvess was implanted as an interposition bypass from the axillary to the mid-brachial artery. Given the extent of soft tissue loss, a muscle flap was placed for coverage. The patient was discharged and was undergoing at-home nursing care including dressings for the wound.

On Day 34, the patient presented with bleeding, in the setting of tissue necrosis over the site of the conduit. Imaging showed irregularities in the vessel, but no pseudoaneurysm or extravasation. On Day 36, the patient experienced a hemorrhage from a rupture in the Symvess graft, which was attributed to open exposure of the conduit due to necrosis of overlying muscle and associated wound infection. Surgical intervention was performed, replacing 70% of the original conduit with a second Symvess segment. Initially, blood flow was restored, but after several procedures to manage the wound, the conduit was no longer pulsatile. Though the distal brachial and ulnar arteries were patent after intervention, the right hand remained ischemic. The patient was noted coagulopathic.

On Day 37, an occlusion of the brachial artery and Symvess necessitated thrombectomy, at which time an iatrogenic injury to the bracial artery was incurred. To resolve this, a portion of the brachial artery was resected, and PTFE was used to bride the resultant gap between the conduit and axillary artery. Though blood flow was achieved through conduit and upper forearm, the hand remained ischemic.

Despite attempts to restore circulation, and on Day 43, due to severe reperfusion injury and extensive infection, a right above-the-elbow amputation was performed. The graft was fully explanted.

Histologic analysis of the explanted Symvess confirmed infection extending from the surrounding tissues into the graft. While infection played a role, the primary cause of the bleeding was determined to be direct, open exposure of the conduit due to breakdown of the necrotic muscle overlying the graft. The subsequent ischemic complications led to the eventual amputation of the limb.

**Case 2**

A 65-year-old male with a history of polysubstance abuse and tobacco use sustained a right leg injury when caught between two cars. Initial examination revealed absent PT/DP pulses bilaterally, an unstable right knee, and signs of vascular compromise. Total ischemia time exceeded 6 hours prior to revascularization. Imaging confirmed occlusion of the right popliteal artery with severe atherosclerosis and stenosis in the distal vasculature. Autologous vein was determined to be of poor quality for repair. Four-compartment fasciotomy precluded the use of PTFE graft.

Symvess was implanted from the distal superficial femoral artery (SFA) to the posterior tibial artery (PTA) on Day 1. However, an initial weak signal and signs of disease in the PTA necessitated a revision of the distal anastomosis for atherectomy in the PTA. The conduit was then re-anastomosed, and hemostasis was achieved. By Day 6, the patient experienced worsening pain and diminished foot perfusion, leading to angiography that confirmed Symvess occlusion. A thrombectomy was performed in the conduit and PTA to restore flow, but angioplasty was required to address PTA stenosis, followed by endarterectomy and patch repair of the PTA. The Symvess was then distally re-anastomosed via hooding over the patch. The soleus muscle that was used to cover the Symvess conduit showed early signs of necrosis but was not deemed nonviable.

On Day 7, the patient developed bleeding from the incision site. Surgical exploration revealed multiple suture failures and a 2mm hole in the conduit, necessitating partial excision and replacement with PTFE. Cultures subsequently confirmed infection. Despite intervention, recurrent thrombosis without a clear cause was observed on Day 8, prompting complete Symvess excision and replacement with a PTFE graft, which also subsequently thrombosed. By Day 17, persistent soft tissue complications and non-viability of the limb led to a right above-knee amputation. The patient was ultimately discharged in stable condition on Day 38.

Histology of the Symvess conduit indicated that there was an external infection in the surrounding tissue, leading to structural compromise. Subsequent PTFE failure was observed in the same location.  This case highlights the multifactorial nature of the failure, where a combination of pre-existing vascular disease, inadequate tissue coverage, and infection contributed to adverse outcomes.

**Case 3**

A 20-year-old male Israeli combat soldier sustained severe bilateral lower extremity injuries after being struck by a car during an operational activity. He presented with open fractures, extensive soft tissue damage, and vascular compromise, scored with a MESS of 6. The right leg exhibited significant displacement of tibia and fibula fractures with absent distal pulses. Autologous vein was not used for repair due to external fixation of both legs, which made vein harvest challenging.

Symvess was implanted for revascularization of the right leg on Day 1. Following Symvess implantation, the orthopedic team had to perform fixator procedures, and fasciotomies of the right leg were performed.  At the end of the surgery, there was still active bleeding from the fasciotomy sites, and it was decided NOT to administer anticoagulant treatment in the coming hours. This patient was classified at high risk of wound infection, due to the contamination of the wounds, the anatomical location of the injury in a limb, the open fractures and external fixators, the crush injuries, the extent of deep tissue involvement, and the overall contamination of the wounds. External fixators were then placed on the left leg, and the wound was debrided.

On the same day, five hours after implantation, the doppler signal, which was excellent after surgery, reportedly disappeared and the foot was cold and pale. The patient was then taken back to the OR, and a thrombectomy was performed.

On Day 17, the patient underwent debridement and a skin graft was placed on the wound over the proximal portion of the conduit, during which time enoxaparin was held. On Day 19, the patient again experienced thrombosis, a thrombectomy was performed, and blood flow to the foot was restored.

On Day 30, an episode of bleeding from the right calf near the vascular repair site occurred. Another bleeding episode occurred on Day 35. Angiography identified extravasation from the proximal portion of the graft under the skin grafted wound, necessitating stent graft placement. On Day 36, the patient experienced Symvess bleeding from the proximal anastomosis. The overlying skin graft that had been covering the ATEV had necrosed, and the infection eroded into the conduit. The Symvess conduit was excised and replaced with an autologous saphenous vein bypass. Despite successful early thrombectomies, infection-related complications and inadequate skin wound coverage ultimately necessitated ATEV removal and autologous vein replacement.

**Case 4**

A 24-year-old male sustained a gunshot wound (GSW) to the left upper chest. He was intubated, received a left-sided chest tube, and had a Foley catheter balloon inserted into the GSW, which appeared to have injured the left subclavian artery. During surgery, he experienced cardiac arrest but was successfully resuscitated. A left upper lobe lung resection and left axillary Argyle shunt placement were performed, and the patient was placed on extracorporeal membrane oxygenation (ECMO).

On the fifth day after sustaining trauma (Day 1 of Symvess placement), the patient underwent definitive repair of the axillary artery injury. Autologous vein was not used as the patient was on ECMO and creating an additional wound was avoided. A 6.5 cm Symvess segment to replace the Argyle shunt was placed as an interposition graft from the left subclavian artery to the left axillary artery, with pulses and Doppler ultrasound signals confirming successful graft placement. By Day 2, cellulitis developed at the left shoulder, requiring vancomycin and cefepime, along with debridement and reclosure of a necrotic wound site.

By Day 10 after Symvess placement, the patient developed a left shoulder wound infection, necessitating surgical debridement and wound vacuum-assisted closure. Cultures confirmed the presence of *Acinetobacter*. Two days later, the patient required a redo thoracotomy for evacuation of a chest wall hematoma and rotational flap placement to cover the Symvess conduit.

On Day 19, there was loss of Symvess primary patency, as the patient bled from the distal anastomosis. Prior to entering the OR, the patient developed an uncontrollable bleed that required emergent Symvess clamping at the bedside. In the OR, intraoperative findings revealed distal anastomotic dehiscence, with intact sutures in the native artery but disruption through the conduit. The conduit was ligated without revascularization due to adequate collateral circulation. The patient recovered from the pseudoaneurysm that same day.

The Symvess conduit was confirmed to be free of tension from the initial operation, and that its dehiscence may have been secondary to maneuvers to clamp the graft in the ICU. Ultimately, severe wound and soft tissue infection were deemed plausibly causal for the formation of the pseudoaneurysm, and the subsequent anastomotic dehiscence leading to Symvess ligation to minimize bleeding.

By Day 23, the patient exhibited no movement or sensation in the left upper extremity, though a duplex scan on Day 24 confirmed arterial flow. On Day 30, thirty-four days from the initial trauma, Doppler ultrasound showed continued distal perfusion; however, motor and sensory deficits persisted. The patient did not require further revascularization due to collateral circulation but experienced persistent left upper extremity deficits. The patient was lost to follow-up after his six-month visit on Day 192 after Symvess placement.
